# The protected flora of long‐established cemeteries in Hungary: Using historical maps in biodiversity conservation

**DOI:** 10.1002/ece3.6476

**Published:** 2020-06-30

**Authors:** Viktor Löki, András Schmotzer, Attila Takács, Kristóf Süveges, Ádám Lovas‐Kiss, Balázs András Lukács, Jácint Tökölyi, Attila Molnár V.

**Affiliations:** ^1^ Wetland Ecology Research Group Centre for Ecological Research, DRI Debrecen Hungary; ^2^ Bükk National Park Wildlife Department Eger Hungary; ^3^ Department of Botany University of Debrecen Debrecen Hungary; ^4^ MTA‐DE “Lendület” Evolutionary Phylogenomics Research Group Debrecen Hungary; ^5^ MTA‐DE Behavioural Ecology Research Group University of Debrecen Debrecen Hungary

**Keywords:** man‐made habitats, nature conservation, red list species, refuges, secondary habitats, vascular plants

## Abstract

The role of anthropogenically influenced habitats in conserving elements of the original wildlife has increased worldwide simultaneously with the disappearance of natural sites. Burial places are able to conserve original elements of the wildlife, and this fact has been known for at least a century. To this day, little is known about long‐time changes and the effect of long‐time management methods in cemeteries on the flora they harbor. The utility of historical maps in research focused on natural values, as well as in answering questions related to conservation was recently demonstrated, but the use of digitized historical maps in biodiversity research of the Carpathian Basin is very limited. In the present paper, we aimed to predict the conservation potential of long‐established and newly established cemeteries of Hungarian settlements with various population sizes based on the digitized maps of the 2nd Military Survey of the Austrian Empire (1819–1869), by categorizing cemeteries into 3 distinct (anthropogenic habitat, cemetery, or natural habitat) types. To build our models, we used records of the protected flora from Hungarian cemeteries, based on data of thematic botanical surveys of 991 cemeteries. Out of the surveyed cemeteries, 553 (56%) harbored protected plants, totaling 306.617 estimated individuals of 92 protected species, belonging to 28 plant families. These species represent 12% of the entire protected flora of Hungary. Hungarian cemeteries play a key role mainly in preserving steppe and dry grassland plant species. Long‐established and large cemeteries harbor more protected plant species than small and newly established ones. Human population size of the settlements correlated negatively with the number of protected species and individuals. Moreover, woodland cover and proportion of grassland also significantly positively affected the number of protected plant species in cemeteries.

## Graphical abstract

The main aim of this paper was predicting the conservation potential of long‐established and newly established cemeteries of Hungarian settlements based on the digitized maps of the 2nd Military Survey of the Austrian Empire (1819–1869), by categorizing cemeteries into 3 distinct (anthropogenic habitat, cemetery, or natural habitat) types. We used records of the protected plant flora from 991 Hungarian cemeteries. 55.80% of surveyed cemeteries harboured protected vascular plants (totally 92 species). Long‐established and larger cemeteries harbour more protected plant species. Human population size of the settlements correlated negatively with the number of protected species and individuals in their cemeteries. Moreover, woodland cover, proportion of grassland, and geographic altitude also significantly affected the number of protected plant species in cemeteries.

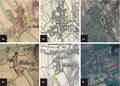

## 1 INTRODUCTION

The surface of Earth has changed drastically due to human activities (Fahrig, 2003). In this transformed landscape, man‐made or human‐influenced habitats serve as refuges for the original wildlife. Such habitats include road verges (Fekete et al., 2017, 2019), midfield islets (Cousins, 2006), and different burial sites, including burial mounds (Deák, Tóthmérész, et al., 2016) or cemeteries, which often preserve the last remnants of natural habitat patches in the transformed urban and/or agricultural landscape. The appreciation of cemeteries in conserving biodiversity has seen an upsurge during the last few decades, and burial sites started to receive special attention in this respect. While studies of the natural values of cemeteries in Europe started with the examination or their bird communities (Pearson, 1915), by the end of the century, further studies were published, revealing the role of cemeteries in the conservation of bryophytes (Fudali, 2001; Mišíková & Kubinská, 2010), lichens (Hawksworth & McManus, 1989; Matwiejuk, 2009), dendroflora (Antkowiak & Heine, 2005), and vascular plants (Czarna, 2016; Šilc, 2009). Nonetheless, to date research on the role of cemeteries in wildlife conservation concentrated to only a few regions of the world (Barrett & Barrett, 2001; Löki, Deák, Lukács, & Molnár V., 2019). Furthermore, we lack thematic surveys that would cover multiple cemeteries in Europe. As such, published papers are geographically strongly biased, and the range of studied organisms is relatively narrow, even in the long‐time‐transformed, densely populated, and well‐researched Europe.

Cultural, ethnographic, and though with less thoroughness but natural values of cemeteries were all deemed noteworthy in the past century in Hungary and in the Carpathian Basin by early works. Nonetheless, despite the valuable historical studies that discuss both the natural and cultural values of Hungarian cemeteries, our knowledge on the role of cemeteries in nature conservation remained sporadic for a long time. In Hungary, ethnographic researchers started to pay greater attention to cemeteries in the second half of the 20th century. For instance, Balassa (1989: 149) suggested that mosaic land use of traditional Hungarian cemeteries, besides the long‐term, traditionally stable, and sustainable management practices of burial places, has favored the conservation of natural habitat patches in the Carpathian Basin: “Hungarian peasant cemeteries usually have three sections. The first section, where no graves had been installed yet, are usually utilised as agricultural plots, where crops are grown and seeds are sown. The second part, usually the largest, where burials take place at the moment and where they took place during the last 35 years. The latter part slowly transits into the third part, where no graves actively cared‐for can be found, and where bushes and trees grow without control, while grass starts to grow on increasingly flat graves. The oldest part of the cemetery is progressively being regained by the natural vegetation.” Consequently, Seléndy (1972) also noted that cemeteries were green fields dominated by grassland vegetation ever since the Middle Ages, and were under protection by various sanctions against nature‐damaging activities for a long time. Novák wrote in 1978 that “Anyone—just like in the case of forests or vineyards—who e.g. caused damage, cut wood, or allowed his/her animals to roam inside the cemetery… was obliged to pay a fine of 12 Hungarian Forints…, the delinquent shall be severely sanctioned, his cows or pigs grazing in the cemetery should be shot without warning.” Cemeteries were regularly hand mown, and the rights to harvesting hay, fruits, and other goods from the cemetery were regularly auctioned until the end of the last century (Balassa, 1989). One of the cemeteries of the Aggtelek‐Karst represents a good example: The successful bidder paid 1,4 EUR for the mowing and harvesting rights in 1977 (Kunt, 1978).

The sustainable management was favorable for the native vegetation of cemeteries in Hungary. Altogether, occurrences of 26 rare plant species were documented in Hungarian cemeteries up to 2000 (Table 1). Moreover, Margittai reported the presence of the extremely rare, endemic *Pulsatilla flavescens* Hazsl. in the cemetery of Cséke (today Lácacséke) (Margittai, 1915). He describes the endangerment of the sandy flora in Cséke and describes the surveyed cemetery as a refuge: “…the cantor's pasture taking up only a couple square yards… was not ploughed because the hillside is rather steep here… even on this soil of running sand a beautiful grassy pasture could have developed. The rare Pulsatilla grows on this small plot and the sandy flora of the surrounding area also found refuge here.”

**TABLE 1:** Vascular plant species protected by Hungarian law detected in Hungarian cemeteries up to 2000

Species	Locality	Year	Collector (herbarium acronym)	Reference
*Festuca wagneri*	Budapest	1869	Dorner (BP)	—
*Dianthus serotinus*	Budapest	1872	Dorner (BP)	—
*Centaurea solstitialis*	Gyula	—	—	borbás (1881)
*Ajuga laxmannii*, *Reseda inodora*, *Taraxacum serotinum*	Makó	—	—	Halász (1889)
*Iris pumila*	Szolnok	1901	Rapaics (bp)	—
*Onosma arenaria*, *Pulsatilla flavescens*	Lácacséke	1911	—	Margittai (1915)
*Linum flavum*	Miskolc	1911	Kiss (bp)	—
*Ranunculus illyricus*	Igrici	—	—	Budai (1914)
*Primula vulgaris*	Győr	1935	Polgár (BP)	—
*Ornithogalum refractum*	Győr	—	—	Polgár (1941)
*Phlomis tuberosa*	Budapest	1944	Siroki (DE)	—
*Ophrys sphegodes*, *Anacamptis morio*, *Neotinea tridentata*	Kaposvár‐Töröcske	1952	—	z. Horváth (2007)
*Scilla vindobonensis*	Csurgósarkad	1954	—	z. Horváth (2007)
*Neotinea tridentata*	Simonfa	1960	—	z. Horváth (2007)
*Neotinea tridentata*	Gálosfa	1960	—	z. Horváth (2007)
*Potentilla rupestris*	Kishuta	1961	Siroki (DE)	—
*Orchis militaris*	Simonfa	1968	—	z. Horváth (2007)
*Astragalus dasyanthus*	Tarcal	1979	Facsar (BP)	Szujkó‐Lacza (1981)
*Thlaspi jankae*	Mezőkövesd	2000	Somlyay (BP)	—
*Ornithogalum brevistylum*	Tótkomlós	—	—	Kertész (2000)
*Amygdalus nana*	Pusztaottlaka	—	—	Kertész (2000)
*Phlomis tuberosa*, *Vinca herbacea*	Kunágota	—	—	Kertész (2000)
*Spiraea crenata*	Pusztamonostor	2000	—	Udvardy (2002)

Later in the North Hungarian Mountains, the flora of cemeteries was surveyed by Schmotzer (2015). He found many valuable plant species, including the endemic *Thlaspi jankae* in 23 cemeteries, a species listed in the Habitats Directive. Moreover, based on the recent field surveys in Hungary, the shrub Spiraea crenata L. previously thought to be extinct in Hungarywas shown to be present in several Hungarian cemeteries (Molnár V., Löki, et al., 2017). Besides the latter species, 26 other valuable and protected plant taxa were identified during the surveys (Molnár V., Löki, et al., 2017).

It has recently been revealed that historical maps can be used to detect long‐term changes in the landscape. They represent useful tools in detecting change in land use (Cui et al., 2014; Skaloš et al., 2011; Skokanová et al., 2012) and vegetation (Kelly, Allen‐Diaz, & Kobzina, 2005), in analyzing the structure of secondary forests (Mladenoff, White, Pastor, & Crow, 1993), or in determining water‐level fluctuations of larger lakes (Zlinszky & Timár, 2013). Historical maps are also appropriate tools for mapping ecosystem services, as well as their changes over a longer period of time (Jiang, Bullock, & Hooftman, 2013). Moreover, historical maps are effective tools in the reconstruction of certain sensitive, disappeared, or shrunken habitats in the landscape (Bromberg & Bertness, 2005). They can also be of help in monitoring the loss and endangerment status of unique habitat types (species listed in the Habitats Directive, Biró, Bölöni, & Molnár, 2018). All in all, historical maps provide an exceptional source of information, providing a glimpse into past and an opportunity to collect data that can be utilized in active nature conservation today. As numerous digitized maps become available for the public, coupled with the development of digitization, and remote sensing techniques, new windows of opportunities open up for nature conservation implications of these historic maps. In Central Europe, the digitized maps of the three (I.‐II.‐III.) Military Surveys of the Habsburg Empire (I: 1763–1787; II: 1819–1869; III: 1869–1887) offer an excellent opportunity for collecting historical information and implementing the above‐mentioned methods in researches of the Carpathian Basin.

The Eurasian forest‐steppe represents one of the most diverse and complex ecosystems in the world, and it stretches from the Ural Mountains on the east to eastern Europe on the west (Erdős et al., 2018). Hungary is situated at the western edge of the Eurasian forest‐steppe ecoregion (Fekete, Molnár, Kun, & Botta‐Dukát, 2002), contributing to the high level of habitat complexity observed in the Pannonian Ecoregion. This unique region harbors many different habitat types on a relatively narrow spatial scale and holds a high level of species diversity (Erdős et al., 2014). Although the Carpathian Basin has an undisputable role in conserving dry grassland and forest‐steppe habitats in Central Europe, most natural habitats have disappeared, and several species have become threatened in recent decades (Molnár, Bölöni, & Horváth, 2008). Consequently, the importance of man‐made habitats in conserving natural elements of the landscape has increased in the new era in parallel with the demand for studies in such unique ecoregions, like the westernmost part of the Eurasian forest‐steppe ecoregion, that would survey these secondary habitats with the goal of identifying and preserving key conservation sites.

In this study, we aimed to carry out a systematic survey of protected plant species in cemeteries that has great conservation potential globally. During our study, we adopted a novel technique: Using historical maps, we aimed to test the predictability of conservation importance of specific burial sites. In detail, we aimed (a) to evaluate which protected plant species are present and how frequently do they occur in Hungarian cemeteries; (b) to test whether long‐established cemeteries host more protected plants than newly established ones; and (c) to determine which are the key characteristics of cemeteries that render them suitable for protected plants. We hypothesized that (1) Hungarian cemeteries host numerous protected plant species in their territory; (2) long‐established cemeteries host more protected plant species than newly established ones; (3) cemetery size, and proportion of grassland and woodland cover will significantly affect the number of plant species and individuals in cemeteries. Our study represents the first systematic survey of the flora in cemeteries of Hungary and the first to implement the use of historic maps in evaluating botanical values of man‐made habitats of the country.

## 2 MATERIALS AND METHODS

### 2.1 Field survey

We surveyed burial grounds between 2014 and 2017, regardless of their religious affiliation, spatial dimension, or geographic location within Hungary. Surveyed cemeteries were selected without any previous knowledge on their history or flora. A total of 991 by a cemeteries were surveyed, including cemeteries from every county of Hungary.

Although the size of cemeteries was not uniform, we endeavored to a complete survey of the sampled cemetery's area; if the area of the cemetery was more than 5 hectares, we focused our searches to reserve areas of cemeteries along the fence. All protected plant taxa were identified and recorded, and the number of individuals belonging to each of these was counted or in the case of more than 500 individuals, estimated in each cemetery. Taxa were identified based on the Plant List (Web 1), and the nomenclature used in this paper also follows this work. Protected plant species were chosen based on the Decree No. 13/2001. (V.9.) of the Ministry of Environmental Protection (Web 2). Geo‐coordinates (WGS84 format) and the elevation (meters above sea level) of the visited cemeteries were determined using a Garmin eTrex Legend handheld GPS device.

### 2.2 Historical maps

In order to identify the age of the surveyed cemeteries, we downloaded historic maps of Hungary, including maps of the First and Second Military Surveys of the Austrian Empire. The digitized maps of the First Military Survey were relatively imprecise and made the localization and georeferencing of cemeteries difficult. Therefore, we disregarded the maps of the First (I.) Military Survey (Web 3) and checked the historical status of surveyed cemeteries based only on the maps of the Second (II.) Military Survey of the Austrian Empire (1806–1869, Web 4). The maps were used to categorize the area of cemeteries. Cemeteries that were already established at the Second Military Survey were considered as long‐established, while those that could not be found on the historic maps were regarded as newly established cemeteries. It is also highly presumable that every settlement had its own burial sites, but as historical maps not always depicted cemeteries at every settlement, we conclude that the lack of cemetery in the close proximity of a given settlement rather means that military surveys considered the smallest cemeteries insignificant for military interests. Therefore, the smallest ones were not indicated on maps; it also follows that if a cemetery was small enough to miss out from the used historical map, then we categorized it to our analysis as newly established, as it probably had a small potential for conserving significant biodiversity in its area. Furthermore, we measured the total area, proportional coverage of woodland vegetation, grassland, graves, and the distance of cemeteries from settlement margins for each surveyed cemetery based on recent satellite images retrieved from the Google Earth Pro software.

### 2.3 Data analyses

Factors influencing the number of protected plant species and the number of individuals of protected species in cemeteries were analyzed using zero‐inflated generalized linear models. Cemetery area (log‐transformed), population size of settlement (log‐transformed), type of site during the 2nd Military Survey (anthropogenic habitat, cemetery, or natural habitat), proportion of area covered by forest, grassland, or graves, and geographic altitude were included as explanatory variables in the model. Models were fitted with both zero‐inflated Poisson (ZIP) and a zero‐inflated negative binomial (ZINB) error distributions, and the better fitting model was selected using Vuong's test. Model selection was applied on full models, and minimum adequate models were obtained using backward stepwise elimination based on the largest p‐values. Statistical analyses were performed in R Statistical and Programing Environment (R Core Team, 2018), using the *pscl* package (Jackman et al., 2017) to perform the zero‐inflated models.

## 3 RESULTS

### 3.1 Number of protected species and individuals

**FIGURE 1:**
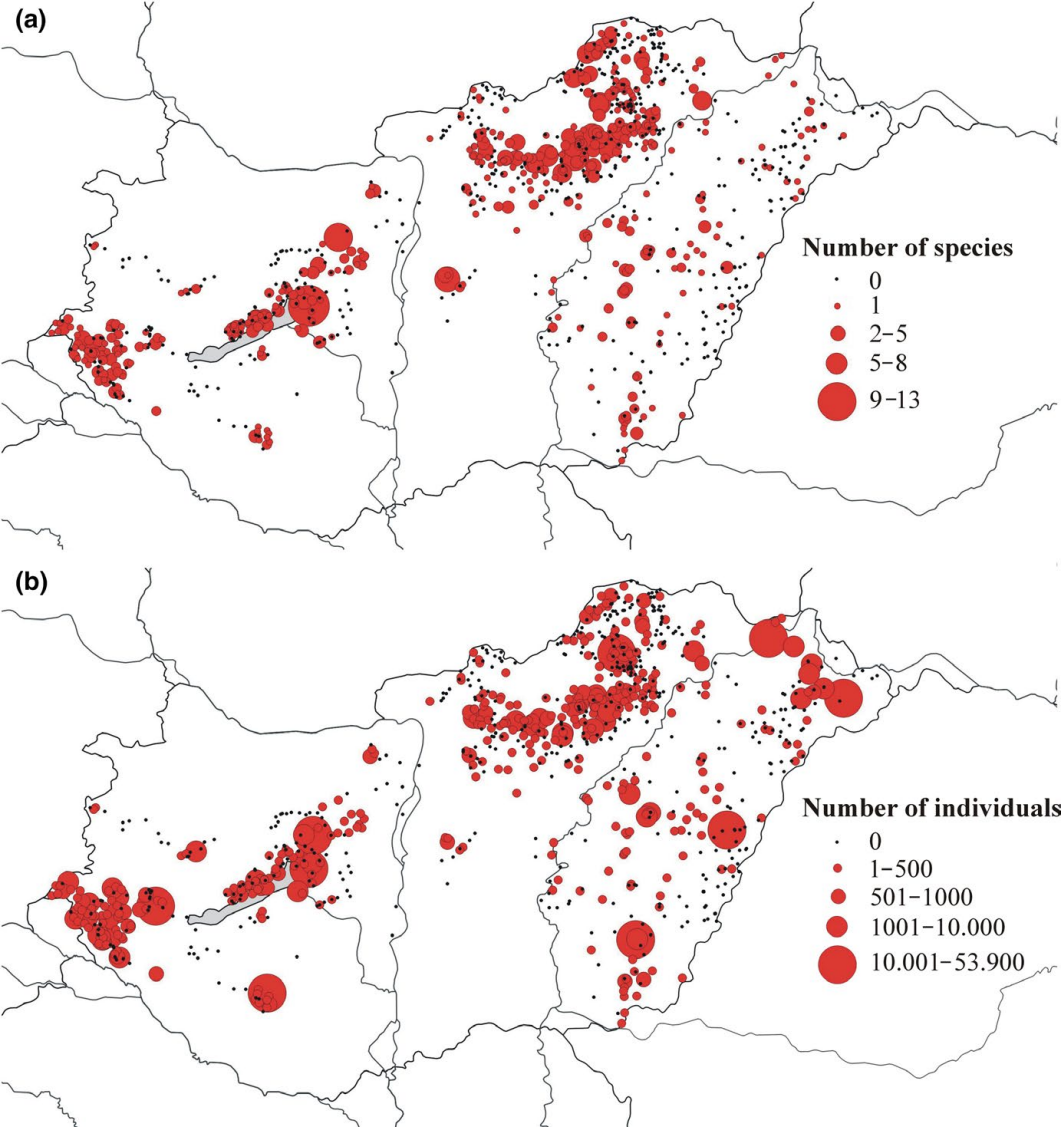
Geographic location, number of found protected species (a), and number of found individuals (b) of the surveyed cemeteries. *N* = 438 cemeteries did not harbor any protected plant species. Red dots represent cemeteries with at least one protected taxa, and black dots represent the ones without. The size of the dots indicates the number of protected plant species and number of individuals

**FIGURE 2:**
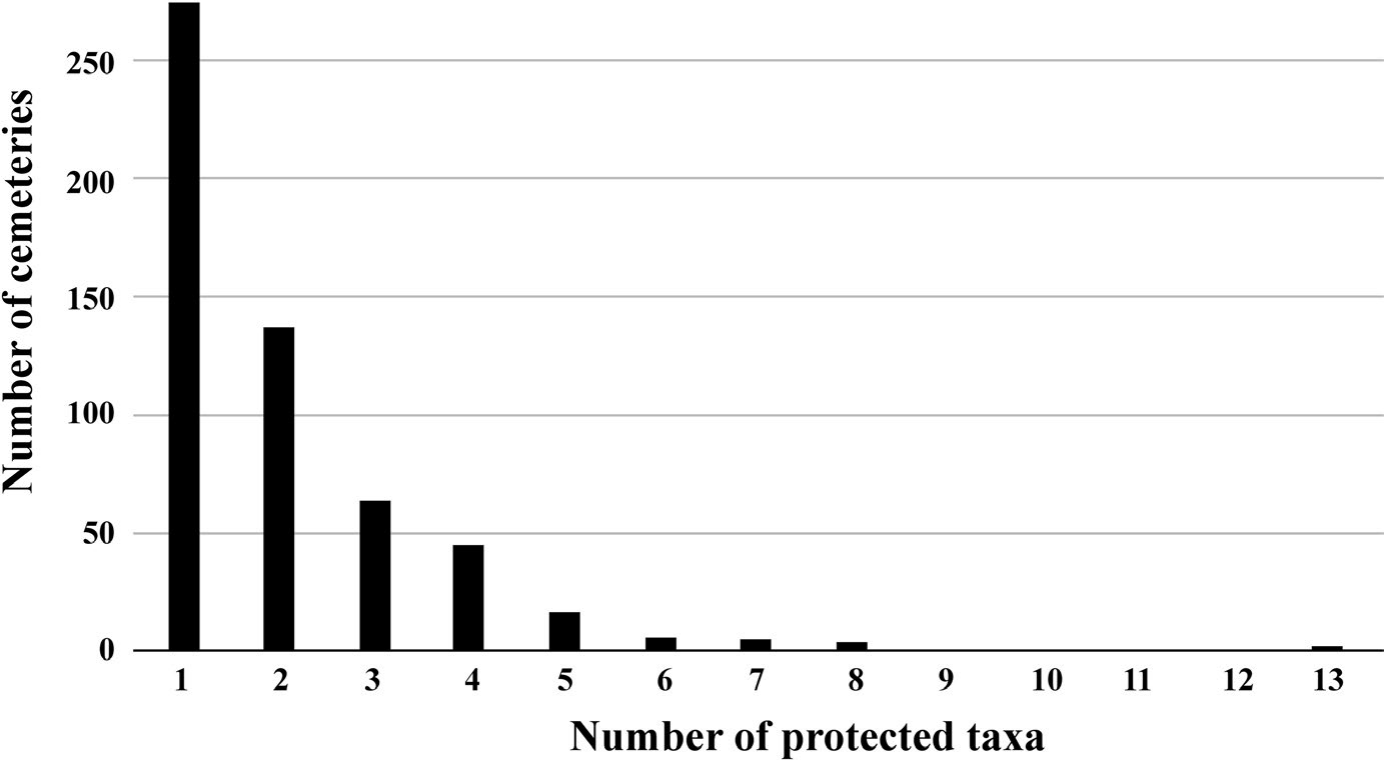
Distribution of the number of protected taxa in the 553 cemeteries harboring at least one taxon protected by the law

553 cemeteries (55.80%) of the evaluated 991 contained at least one protected plant species (Figure 1.). The number of protected plant species occurring in a single cemetery varied from one to thirteen (mean ± *SD* = .12 ± 1.47). 275 cemeteries hosted one, 277 cemeteries harbored 2–8, while one cemetery harbored an exceptional 13 protected taxa (Figure 2.). 41 protected taxa were found in only one of the 991 surveyed cemeteries. The number of protected plants found in more than ten cemeteries was 18. The most abundant five species were *Amygdalus nana* (91 cemeteries, this species is also frequently planted in cemeteries), *Ornithogalum brevistylum* (76 cemeteries), *Ranunculus illyricus* (72 cemeteries), *Thlaspi jankae* (69 cemeteries), and *Phlomis tuberosa* (55 cemeteries). The family Orchidaceae had a disproportionately high representation in cemeteries: A total of 12 orchid species were found in 80 cemeteries, with an estimated number of 7.378 individuals. The most populous orchid in Hungarian cemeteries was *Anacamptis morio*, with 6.696 individuals in 53 distinct populations.

**FIGURE 3:**
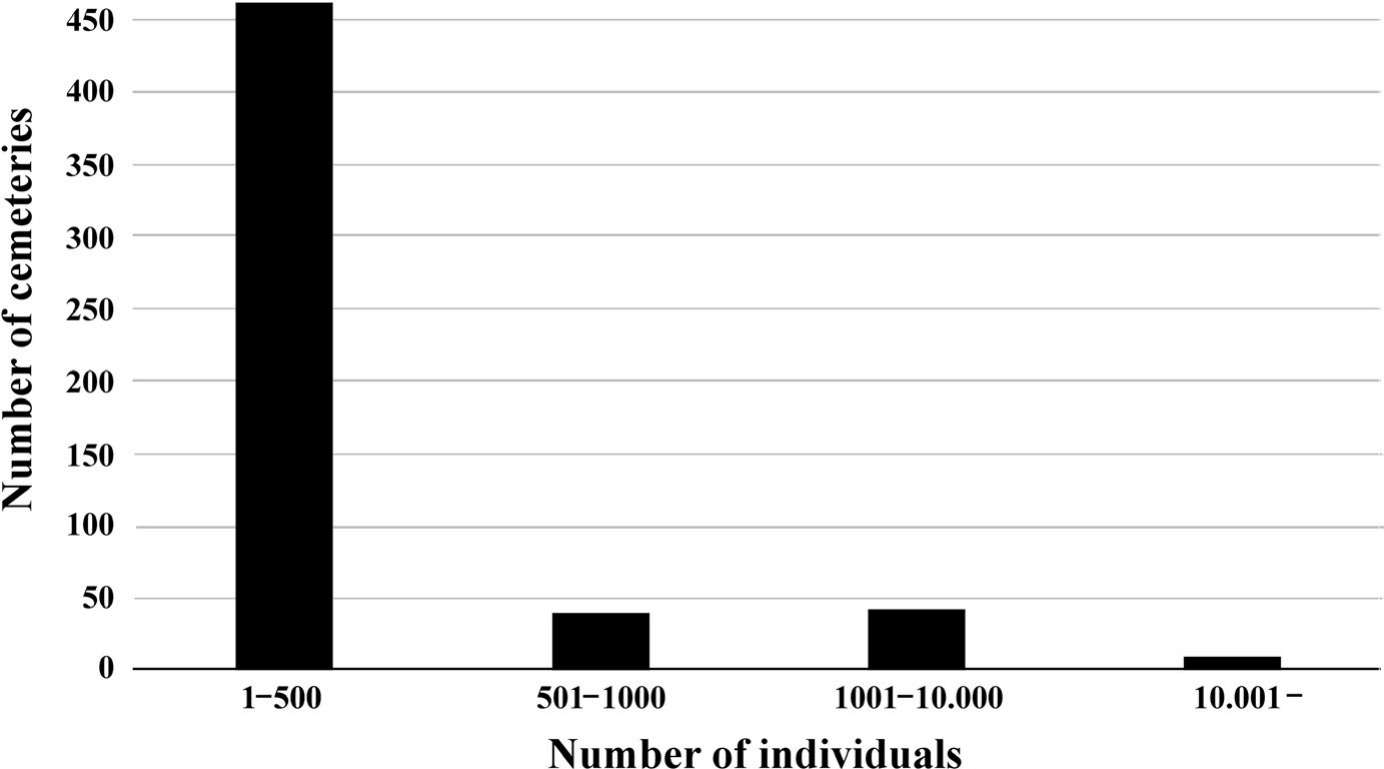
Number of individual plants belonging to protected taxa in the 553 cemeteries harboring at least one species protected by the law. *N* = 438 cemeteries did not harbor any protected plant species

**FIGURE 4:**
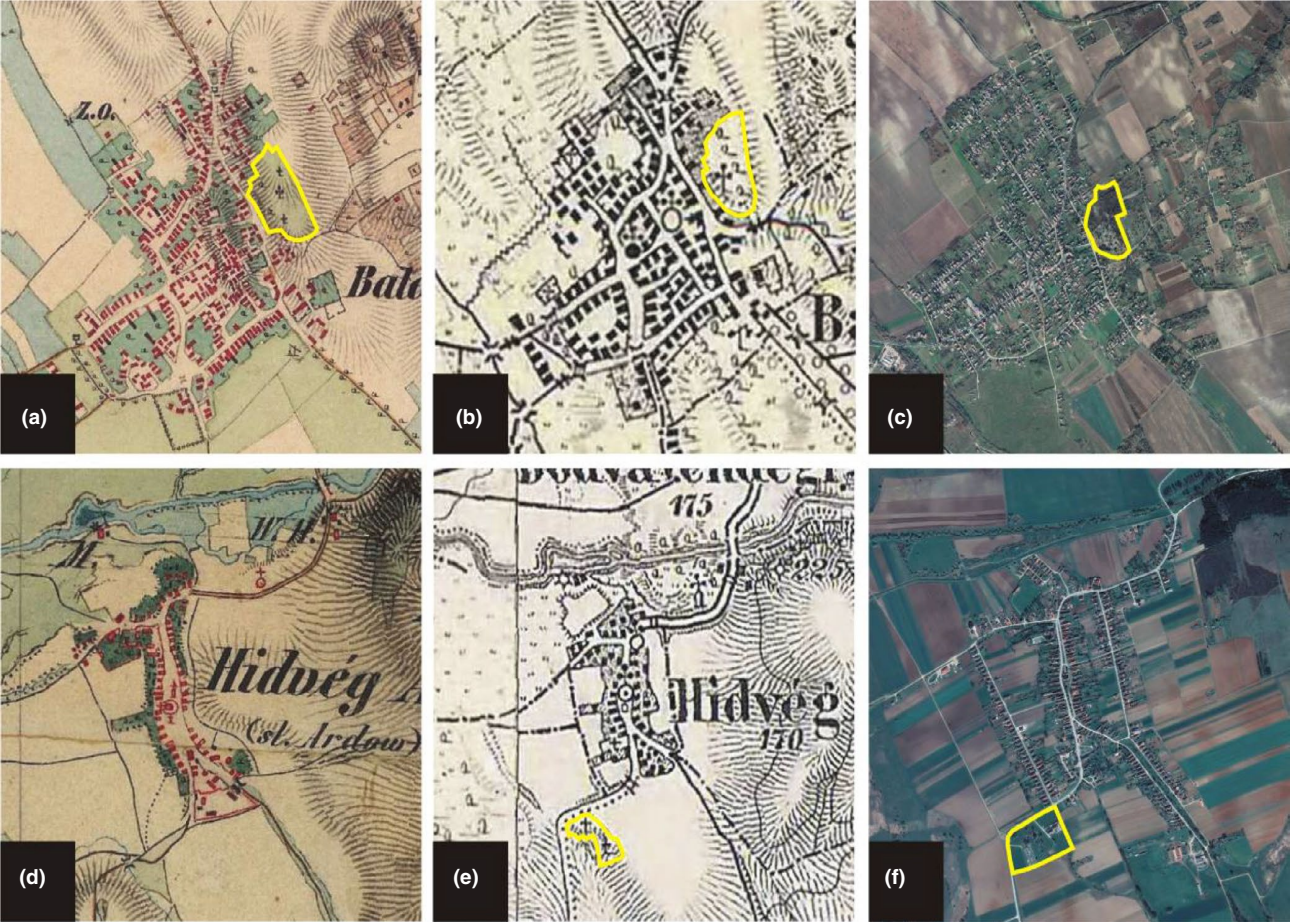
Cemeteries of Balatonfőkajár (a–c) and Hídvégardó (d–f) in the past 200 years on historical maps, and on a recent Google Earth satellite image. Yellow polygons represent the area of cemetery. The cemetery of Balatonfőkajár (a–c) harbors the highest number of protected plant species in Hungary among the surveyed cemeteries. (a) Map detail of the Second Military Survey. (b) Map detail of the Third Military Survey. (c) Map detail of a recent Google Earth satellite image. Image taken on 06/11/2018. (d) Map detail of the Second Military Survey (according to the survey, the cemetery was only established later). (e) Map detail of the Third Military Survey (where the cemetery already present). (f) Map detail of a recent Google Earth satellite image. Image taken on 06/11/2018

The number of plant individuals belonging to protected species detected in a single cemetery varied from one to 53.900 (mean ± *SD* = 316.48 ± 2,248.35, Figure 4.). The number of protected taxa with over 1,000 individuals present in the surveyed cemeteries was 17. Cemeteries with extremely high number of individuals belonging to protected plant species are quite rare in Hungary: The vast majority of surveyed cemeteries hosted 1–500 individuals. The number of cemeteries harboring more than 1,000 individuals belonging to protected taxa was 51 (5.34%), while only nine cemeteries hosted over 10.000 individuals of protected plants (Figure 3). Individual‐rich cemeteries (with more than 1,000 individuals of protected plants) were mostly located in the mountainous region of Hungary; however, some significant populations of protected plants also occurred at lowlands (Figure 1), some harboring more than 10.000 individuals. A total of 306.617 estimated individuals of 92 plant taxa protected by the Hungarian law were detected in cemeteries within the framework of this study (Table S1).

### 3.2 The age of cemeteries and their conservation potential

The historical habitat type of the cemeteries significantly affected the number of species they currently harbor. Long‐established cemeteries hosted more protected plant individuals than newly established ones (categorized as other habitat types on the historical maps, Figure 4.), while no correlation was found between historical habitat type and the number of protected plant individuals present.

### 3.3 Factors affecting species richness and abundance

**FIGURE 5:**
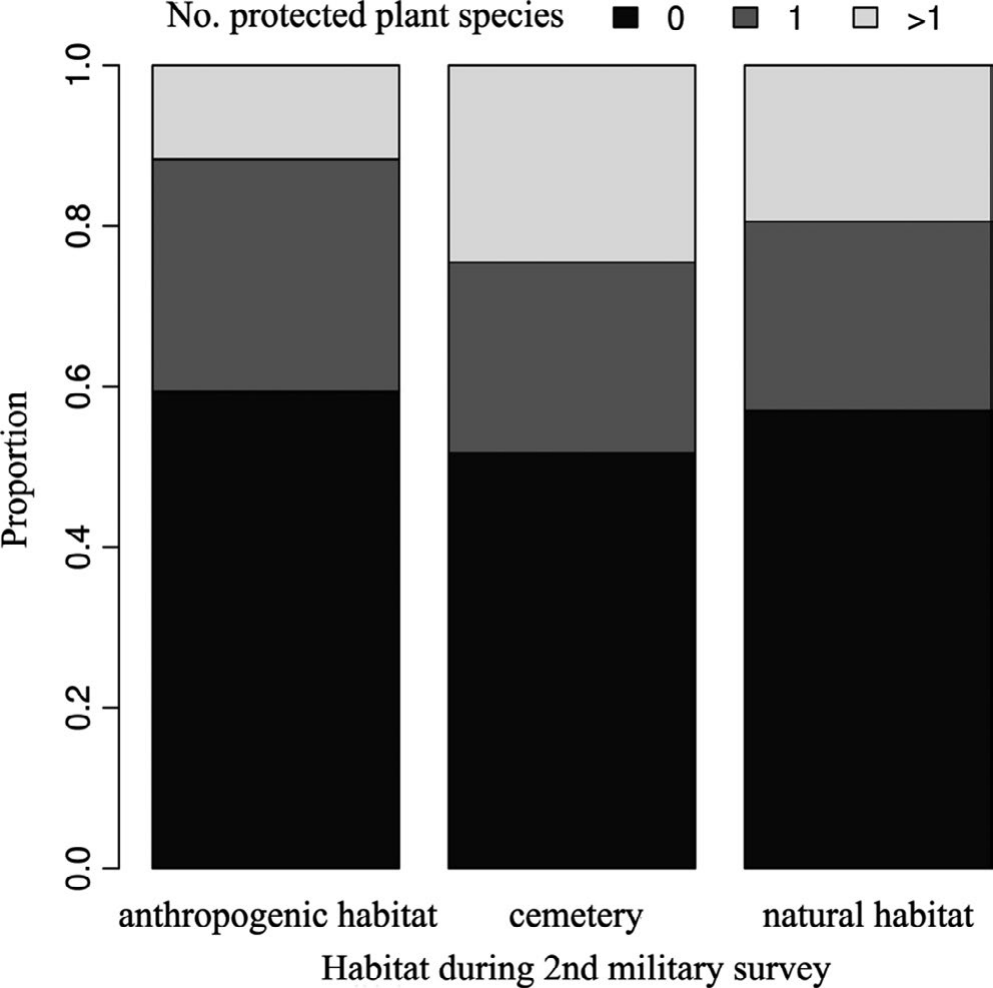
Long‐established cemeteries host more protected plant species than those converted from anthropogenic habitats into cemeteries relatively recently (i.e., after the 2nd Military Survey)

The ZIP and ZINB models built to explain the number of protected species were not significantly different from each other based on Vuong's test (*z* = −1.54, *p* = .062); hence, we selected the simpler ZIP model. Based on the minimally adequate ZIP model, the number of protected plant species in cemeteries was negatively related to settlement population size and to the proportion of area covered by graves in the cemetery, and positively related to the proportion of area covered by grassland and forest (Table 2.). Long‐established cemeteries hosted significantly more protected plant species than cemeteries converted from anthropogenic habitats, while those converted from natural habitats did not differ significantly from cemeteries converted from anthropogenic habitats (Table 2 and Figure 5).

**TABLE 2:** Factors explaining variation in the number of protected plant species in Hungarian cemeteries, as inferred from zero‐inflated Poisson generalized linear models

Parameter	Full model		Minimal model	
	Estimate (*SE*)	*p*‐Value	Estimate (*SE*)	*p*‐Value
Cemetery area (log‐transformed)	0.262 (0.070)	<.001	0.240 (0.064)	<.001
Settlement population size (log‐transformed)	−0.127 (0.041)	.042	−0.114 (0.037)	.002
2nd Military Survey habitat type (cemetery vs. anthropogenic habitat)	0.349 (0.123)	.004	0.357 (0.121)	.003
2nd Military Survey habitat type (natural vs. anthropogenic habitat)	0.180 (0.170)	.288	0.153 (0.356)	.346
Proportion of area covered by forest	1.203 (0.354)	<.001	1.200 (0.282)	<.001
Proportion of area covered by grassland	0.795 (0.215)	<.001	0.799 (0.207)	<.001
Proportion of area covered by graves	−0.094 (0.234)	.010		
Altitude	0.002 (0.001)	.007	0.003 (0.001)	.001

In case of individual numbers of protected plants, Vuong's test indicated a statistically better fit of a ZINB model compared with the ZIP model (*z* = −14.77, *p* < .001); hence, the former were used. Based on the minimally adequate ZINB model, the number of individuals of protected plant species was positively related to cemetery area and negatively related to the proportion of area covered by forests and graves (Table 3.).

**TABLE 3:** Factors explaining variation in the number of individuals of protected plant species in Hungarian cemeteries, as inferred from zero‐inflated negative binomial generalized linear models

Parameter	Full model		Minimal model	
	Estimate (*SE*)	*p*‐Value	Estimate (*SE*)	*p*‐Value
Cemetery area (log‐transformed)	0.884 (0.250)	<.001	0.400 (0.165)	.037
Settlement population size (log‐transformed)	−0.275 (0.132)	.037		
2nd Military Survey habitat type (cemetery vs. anthropogenic habitat)	0.631 (0.394)	.112		
2nd Military Survey habitat type (natural vs. anthropogenic habitat)	0.763 (0.519)	.142		
Proportion of area covered by forest	−4.171 (1.088)	<.001	−2.824 (0.774)	<.001
Proportion of area covered by grassland	−1.068 (0.758)	.078		
Proportion of area covered by graves	−2.873 (0.789)	<.001	−3.055 (0.700)	<.001
Altitude	0.003 (0.003)	.359		

## 4 DISCUSSION

The current paper presents the first thematic field survey of the protected vascular flora of Hungarian cemeteries. Protected plants were found in 55.80% of the 991 evaluated cemeteries. The 92 protected plant species detected in cemeteries represent the 12.43% of the protected vascular [732 species, via Decree No. 13/2001. (V.9.), Web 2], and the 4.17% of the total native flora (2,180 species based on the work of Horváth et al., 1995) of Hungary. Therefore, we suggest that cemeteries represent key habitats of rare plants in Hungary, similarly to what has been demonstrated in some other countries (e.g., Buchholz et al., 2016; Kowarik & Buchholz, 2016; Löki, Deák, et al., 2019; Löki, Molnár V., et al., 2019; Löki et al., 2015; Molnár V., Löki, et al., 2017; Molnár V., Nagy, et al., 2017; Molnár V., Süveges, Molnár V., & Löki, 2017; Molnár V., Takács, et al., 2017).

Although nowadays the land use of cemeteries is changing rapidly, burial places still harbor significant plant diversity. Based on the species compositions described here, it seems that cemeteries play a key role in conserving mainly steppe (Molnár V., Löki, et al., 2017) and dry grassland (Molnár V., Mészáros et al., 2018) plant species. This pattern can also be attributed to the fact that an overwhelming proportion of protected plant species in Hungary are steppic or dry grassland species.

Our results also highlight that the conservation potential of cemeteries varies widely across different regions of the country. This pattern can be attributed to both biogeographic patterns and land‐use factors. Similarly to most places in the world, at the time of the establishment the appropriate place for the cemeteries in Hungary was selected based on practical, for example, security and religious considerations (Seléndy, 1972:19), while esthetics was only a secondary criterion. The dynamic mosaic structure of traditional cemeteries mentioned by Balassa (1989) proved to be a sustainable management method for centuries. The cemetery remained a part of nature, but people could moderately alter the cemetery for both burial and extensive economic purposes (e.g., orchards and hay meadows), while in parallel with these, excessive nature destructive actions were strictly forbidden in most cemeteries (Novák, 1978).

Historical data retrieved from old maps indicated that the number of habitats of a given territory can decrease significantly over time (Jiang et al., 2013). Another study that used historical maps to reconstruct past conditions showed that the semi‐natural grassland cover decreased by over 96% in the studied area since the beginning of the 20th century in Sweden (Cousins, Auffret, Lindgren, & Tränk, 2015). An excellent study has been conducted about habitat loss in Hungary by evaluating 5,000 randomly selected localities over 7 periods from 1783 to 2013 (Biró et al., 2018): According to this study, trends in habitat loss over time are strongly habitat‐specific, and most habitats had continuous loss from period to period. As a consequence, cemeteries are currently emerging as small islands that conserve natural and semi‐natural grasslands in the radically changed landscape, as indicated by the high number of protected grassland and steppic plant species detected in Hungarian cemeteries. However, it must be added that they are not exclusively the guardians of grassland and steppic organisms in the close proximity of urban areas: According to the findings of Deák, Valkó et al. (2020), burial mounds (kurgans) also play a key role in conserving at least grassland specialist plants and arthropod species, while their potential in conserving other organisms is also presumably high.

Our results indicate that long‐established cemeteries harbored significantly more protected plant species than newly established ones. This result supports the hypothesis that cemeteries represent habitat patches that more or less preserved their natural states, and provide safe harbors for natural habitats (and their protected organisms). These habitats remained only slightly disturbed over time and provided colonization windows for rare and sensitive organisms, and also provided a kind of micromosaicism in the habitat. It is very important to note that long‐established cemeteries did not host more protected plant individuals than newly established ones. We believe that this lack of association might indicate the effect of incorrect management practices. Although old cemeteries have higher conservation potential, due to their higher biodiversity, especially with respect to protected species, their incorrect management might keep these species at bay and prevent their population growth. On the other hand, cemetery area has been shown to positively correlate with the number of protected individuals they harbor. Consequently, the species composition appears to be more representative of the conservation potential of a cemetery, than the number of individuals belonging to protected taxa found in them. This pattern might emerge due to the fact that many years of improper management is necessary to entirely eliminate a species from a population, but a high proportion of individuals can be eradicated with only a few years of intensive management, especially in the case of grasslands (e.g., Plantureux, Peeters, & McCracken, 2005).

We found that more protected plant species can be found in cemeteries at higher altitudes (Figure 1). This pattern might emerge from the different species pools of different climatic zones at different altitudes. However, according to our personal observations, radical transformation of the landscape, frequent use of herbicides, and use of motorized mowing devices are more frequent in lowland cemeteries, rendering these less suitable for rare and sensitive organisms. Moreover, lowland areas contain more settlements with higher number of inhabitants, which can explain the more intensive management adopted in lowland cemeteries and the concentration of rare organisms at higher altitudes.

The total area of cemeteries positively correlated with the number of protected individuals found in them. This result suggests that the size of cemeteries can significantly influence their natural values. However, this does not necessarily mean that small cemeteries have small potential for conserving natural values, as many surveyed cemeteries which were small or average in area harbored important botanical values. Deák, Valkó, Török, and Tóthmérész (2016) found the same in case of ancient burial mounds: The number of grassland specialist species was not influenced by the area of the burial mound. They concluded that the main reason for that was the increased level of environmental heterogeneity; this might be also true in the case of cemeteries. The prominent role of cemeteries of large spatial extent in conserving botanical values suggests that these sites can play key roles in the drastically changed landscapes; while it seems regardless of their size, they also represent small natural islands that might serve as key stepping stones for certain organisms.

The human population size of settlements negatively correlated with the number of protected plant species and the number of individuals belonging to these. Consequently, cemeteries of small settlements have the greatest potential to conserve protected plants in Hungary. Therefore, we suggest that the greatest attention of both professional conservation biologists and local people should be devoted to cemeteries of small villages. Given that most sites harboring species of conservation concerns are exempt from nationwide level protection, we press that these cemeteries should urgently receive protection at least on the regional level. Our observations are in consent with previous field surveys conducted in Turkey (Löki, Molnár V., et al., 2019), where one of the key factors explaining the conservation value of cemeteries was the type (village, small town, or city) of the nearby human settlement. The latter study suggested that urbanization is a key threatening factor to natural values in cemeteries. We believe that it is a pressing need to identify newly adopted management practices of cemeteries that are unsustainable for the conservation of natural values on the long term.

In parallel with the change in traditional management practices, the structure of the flora has also changed in Hungarian cemeteries over the past decades (Figure 4). During our field surveys, we documented invasion by some highly invasive plant taxa (i.e., *Robinia pseudo‐acacia*, *Ailanthus altissima*, *Syringa vulgaris*, *Solidago* spp., *Fallopia* spp.), but historical records also indicate that some plants regularly invaded cemeteries in the past. For instance, *Robinia pseudo‐acacia*, *Hedera helix*, and *Syringa vulgaris* were previously mentioned to occur in Hungarian cemeteries, often as widespread species that radically transform the landscape of burial places (Balassa, 1989).

The religious affiliation of cemeteries is considered to be a key factor affecting the landscape and wildlife diversity in their territory (see Molnár V., Takács, et al., 2017). According to ethnographic researchers, Calvinist cemeteries are less cared compared with Catholic ones (Balassa, 1989), also reflected by the lower frequency and number of ornamental plants in the former. Accordingly, some ethnographers concluded that Calvinist cemeteries are more neglected (Balassa, 1989:149), while detailed analyses need to be applied to compare the effects of religious affiliation on natural values in more European cemeteries.

Novel cemetery management practices are highly divergent from traditional ones, and a “well‐maintained” cemetery implies intensified pressure on the natural vegetation (e.g., motorized mowing, herbicides, use of concrete). Modern gardeners usually favor low‐mowed lawns. For example, a Hungarian cemetery managing handbook recommends that if the grass reaches 8 cm in height the area should be mowed (Seléndy, 1972:224). Nonetheless, Hungarian ethnographers, historians, suggest that the extremely low, monodominant vegetation is unanimous, just like the modern cemetery landscape, dominated by high density, large concrete graves (Pocsai & Tóth, 1990:8). We suggest that hand mowing would be the most appropriate method in order to preserve these grassland habitats the most effectively, while reducing the number of electric mowings in most of the areas would provide almost the same effectiveness in preserving most of the sensitive species.

Percentage of woodland and grassland cover of the vegetation positively affected the number of protected plant species found in cemeteries. Although large areas of grasslands can provide shelter for protected plant species, the stability and long‐term survival of these populations is severely affected by the management methods. Large areas of grassland might still be unable to support protected plant species if the grass is mowed too often. This is further supported by the negative correlation of the area covered by graves in the cemetery and the number of protected plant individuals: Although plants sometimes survive among graves, the optimal habitat patches for them are semi‐natural, unfragmented areas that are usually only found on the periphery of cemeteries. Graves are usually uncolonizable due to their artificial material or their intensive management. Moreover, the traditional use of cemeteries for harvesting hay or fruits is discontinued in most regions of the world. Losing the need for ecosystem services of cemeteries, such as cutting of cemetery orchards or changing forests and woody vegetation in cemeteries for non‐native trees and forests, can also result in a significant loss in biodiversity: According to Löki, Molnár V., et al. (2019), conservation value was positively related to proportion of native trees in cemeteries of Turkey. However, mostly grassland cemeteries are occurring in Hungary, but as the woodland cover was also positively related to the number of protected species in cemeteries, the important role of woodland vegetation in creating mosaic habitats in cemeteries is still inevitable.

Afforested cemeteries are well‐functioning, while also representing valuable and esthetic parks, providing potential recreational benefits for people, especially in the urban landscape. However, such cemeteries cannot be realized on the long run, if the modern burial protocols continue to be adopted and implemented with intensities seen during the last century. “We shall not look for immortality in cold and lifeless stones” (Pocsai & Tóth, 1990:10). Instead, we should adopt new and progressive ways to rejuvenate the area of cemeteries. For instance, according to Holden and McDonald‐Madden (2018) conservation burials are carried out in the highest numbers in the UK, the United States, and Canada. The fees paid for these burials are used to fund the acquisition, protection, restoration, and management of lands that benefits human and environmental well‐being.

We suggest that further studies should be conducted to identify the role of cultural factors and religious affiliations in determining the conservation value of cemeteries. Our results also suggest that thematic surveys of cemeteries that concentrate on the detection of multiple taxa, especially on a wide geographic scale, would provide valuable information on the role of anthropogenically influenced habitats in conserving valuable habitats and sensitive or rare organisms. Based on this systematic survey, we conclude that historical maps provide an excellent opportunity in biodiversity conservation to effectively designate sampling areas with presumably high conservation value for further research.

## CONFLICT OF INTEREST

None declared.

## AUTHOR CONTRIBUTION

**Viktor Löki:** Investigation (equal); Methodology (equal); Writing‐original draft (equal); Writing‐review & editing (equal). **András Schmotzer:** Investigation (equal); Writing‐review & editing (equal). **Attila Takács:** Investigation (equal); Writing‐review & editing (equal). **Kristóf Süveges:** Investigation (equal); Writing‐review & editing (equal). **Ádám Lovas‐Kiss:** Investigation (equal); Writing‐review & editing (equal). **Balázs A. Lukács:** Investigation (equal); Writing‐review & editing (equal). **Jácint Tökölyi:** Data curation (equal); Formal analysis (equal); Methodology (equal); Writing‐original draft (equal); Writing‐review & editing (equal). **V. Attila Molnár:** Conceptualization (equal); Funding acquisition (equal); Investigation (equal); Methodology (equal); Supervision (equal); Writing‐original draft (equal); Writing‐review & editing (equal).

## WEB REFERENCES

Web 1: The Plant List (2013): Version 1.1. http://www.theplantlist.org/ [accessed: 2018.03.05.]
Web 2: 100/2012 (IX. 28.) VM decree of protected and highly protected species of Hungary. – Magyar Közlöny 128: 20903–21019. http://nmhh.hu/dokumentum/153733/mk_12_128.pdf [accessed: 2018.03.05.]
Web 3: Digitised map sheets of the 1st Military Survey of the Austrian Empire http://mapire.eu/en/map/firstsurvey/ [accessed: 2018.03.05.]
Web 4: Digitised map sheets of the 2nd Military Survey of the Austrian Empire http://mapire.eu/en/map/secondsurvey/ [accessed: 2018.03.05.]

## Supporting information

Appendix S1

## Data Availability

All data used in the analyses are available from Dryad—https://doi.org/10.5061/dryad.jwstqjq6c (Löki et al., 2020).
