# Supplementary material for: The protected flora of long‐established cemeteries in Hungary: Using historical maps in biodiversity conservation
Source: Ecol Evol. 2020 Jun 30;10(14):7497–508. doi: 10.1002/ece3.6476 (PMC7391536; doi:10.1002/ece3.6476)
Supplement: Supplementary file 1 — Appendix S1 [file ECE3-10-7497-s001.docx]

**Electronic appendix**

Table S1. Protected plant species found in Hungarian cemeteries

| **Protected taxon** | **Distribution** | **Total number of populations** | **Total number of individuals** |
| --- | --- | --- | --- |
| *Amygdalus nana* L. | Pontic | 91 | 727 |
| *Ornithogalum brevistylum* Wolfner | Mediterranean | 76 | 13.008 |
| *Ranunculus illyricus* L. | Pontic-Mediterranean | 72 | 46.183 |
| *Thlaspi jankae* A. Kern. | Pannonian | 69 | 25.726 |
| *Phlomis tuberosa* L. | Eurasian | 55 | 6696 |
| *Anacamptis morio* (L.) R.M.Bateman, Pridgeon & M.W.Chase | Central-European | 53 | 5176 |
| *Primula vulgaris* Huds. | Asian-Mediterranean | 50 | 22.835 |
| *Clematis integrifolia* L. | Continental | 33 | 2175 |
| *Vinca herbacea* Waldst. & Kit. | Pontic-Pannonian | 29 | 1624 |
| *Sternbergia colchiciflora* Waldst. & Kit. | East-Submediterranean | 27 | 45.785 |
| *Adonis vernalis* L. |  | 20 | 3176 |
| *Taraxacum serotinum* (Waldst. & Kit.) Fisch. | Pontic-Pannonian | 19 | 3802 |
| *Linaria biebersteinii* Besser | Eurasian | 19 | 1216 |
| *Spiranthes spiralis* (L.) Chevall. | Continental | 19 | 795 |
| *Galanthus nivalis* L. | Central-European | 17 | 74.163 |
| *Spiraea crenata* L. | Continental | 14 | 26 |
| *Neotinea tridentata* (Scop.) R.M.Bateman, Pridgeon & M.W.Chase | South-Mediterranean | 13 | 760 |
| *Iris pumila* L. | Pontic-Pannonian | 12 | 27 |
| *Orchis purpurea* Huds. | Central-European | 10 | 189 |
| *Scilla vindobonensis* Speta | Central-European | 8 | 32.761 |
| *Aster sedifolius* L. | Continental | 8 | 8859 |
| *Polygala major* Jacq. | Pontic-Mediterranean | 8 | 756 |
| *Ornithogalum refractum* Kit. ex Schltdl. | Balcanic | 7 | 31 |
| *Lychnis coronaria* Desr. | South-Mediterranean | 5 | 52 |
| *Muscari botryoides* (L.) Mill. | Pontic-Mediterranean | 4 | 786 |
| *Aster amellus* L. | Pontic | 4 | 253 |
| *Potentilla patula* Waldst. & Kit. | Pontic-Pannonian | 4 | 186 |
| *Stipa sp.* | N/A | 4 | 115 |
| *Scilla drunensis* (Speta) Speta | N/A | 4 | 114 |
| *Iris aphylla* L. subsp. *hungarica* Hegi | Pannonian | 4 | 33 |
| *Dictamnus albus* L. | Pontic-Mediterranean | 4 | 23 |
| *Festuca wagneri* (Degen, Thaisz & Flatt) Krajina | Pannonian | 3 | 300 |
| *Centaurea arenaria* M.Bieb. ex Willd. | Pontic-Pannonian | 3 | 290 |
| *Inula oculus-christii* L. | Pontic-Pannonian | 3 | 216 |
| *Pulsatilla grandis* Wend. | Pontic-Pannonian | 3 | 202 |
| *Doronicum hungaricum* (Sadler) Rchb.f*.* | Pannonian-Balcanic | 3 | 170 |
| *Anthriscus nitidus* (Wahlenb.) Hazsl. | Central-European-Alpine | 3 | 122 |
| *Centaurea sadleriana* Janka | Pannonian | 3 | 27 |
| *Carlina acaulis* L. | Central-European | 3 | 26 |
| *Iris variegata* L. | Pontic-Pannonian | 3 | 5 |
| *Medicago rigidula* (L.) All. | Mediterranean | 3 | 3 |
| *Prunella grandiflora* (L.) Scholler | European | 2 | 550 |
| *Iris arenaria* Waldst. & Kit. | Pannonian | 2 | 180 |
| *Silene bupleuroides* L. | Pontic | 2 | 110 |
| *Cephalanthera damasonium* (Mill.) Druce | Central-European | 2 | 101 |
| *Dianthus collinus* Waldst. & Kit. | South-Mediterranean | 2 | 60 |
| *Convolvulus cantabrica* L. | South-Mediterranean | 2 | 51 |
| *Centaurea triumfetti* All. | Eurasian | 2 | 16 |
| *Epipactis helleborine* (L.) Crantz | Eurasian | 2 | 12 |
| *Pulsatilla nigricans* Storck | Central-European | 2 | 8 |
| *Inula germanica* L. | Pontic-Pannonian | 2 | 3 |
| *Peucedanum officinale* L. | Central-European | 1 | 5000 |
| *Scilla autumnalis* L. | South-Mediterranean | 1 | 500 |
| *Astragalus excapus* L. | Central-European | 1 | 400 |
| *Anacamptis pyramidalis* (L.) Rich. | South-Mediterranean | 1 | 150 |
| *Ophrys sphegodes* Mill. | South-Mediterranean | 1 | 140 |
| *Scopolia carniolica* (Jacq.) Kuntze | Carpathian-Endemic | 1 | 100 |
| *Achillea ochroleuca* Ehrh. | Pontic-Pannonian | 1 | 100 |
| *Stipa borysthenica* Prokudin | Pontic-Pannonian | 1 | 100 |
| *Scabiosa canescens* Waldst. & Kit. | Central-European | 1 | 100 |
| *Ophrys fuciflora* (F.W.Schmidt) Moench | Asio-Mediterranean | 1 | 50 |
| *Astragalus dasyanthus* Pall. | Pontic-Pannonian | 1 | 22 |
| *Ranunculus polyphyllus* Waldst. & Kit. ex Willd. | Continental | 1 | 20 |
| *Potentilla rupestris* L. | Circumpolar | 1 | 15 |
| *Jovibarba hirta* (Pollini) Opiz | Central-European-Alpine | 1 | 10 |
| *Reseda inodora* Rchb. | Pontic-Pannonian | 1 | 5 |
| *Cirsium erisithales* (Jacq.) Scop. | Central-European | 1 | 5 |
| *Spiraea media* Schmidt | Continental | 1 | 5 |
| *Echium russicum* J.F. Gmel. | Pontic-Pannonian | 1 | 4 |
| *Thalictrum aquilegifolium* L. | European | 1 | 4 |
| *Primula elatior* (L.) Hill | Eurasian | 1 | 3 |
| *Pulsatilla Zimmermannii Soó* | Pannonian | 1 | 3 |
| *Pyrola rotundifolia* L. | Circumpolar | 1 | 3 |
| *Cephalanthera longifolia* (L.) Fritsch | European | 1 | 2 |
| *Neottia nidus-avis* (L.) Rich. | Eurasian | 1 | 2 |
| *Sempervirum marmoreum* Griseb. subsp. *matricum* Letz | Pannonian | 1 | 2 |
| *Silene dioica* (L.) Clairv. | European | 1 | 2 |
| *Himantoglossum adriaticum* H.Baumann | Atlantic-Submediterranean | 1 | 1 |
| *Polystichum aculeatum* (L.) Roth ex Mert. | Eurasian | 1 | 1 |
| *Lathyrus lacteus* (M. Bieb.) E.D. Wissjul. | Pannonian | 1 | 1 |
| *Achillea chritmifolia* Waldst. Et Kit. | Pannonian-Balcanic | 1 | 1 |
| *Dryopteris carthusiana* (Vill.) H.P. Fuchs | Circumpolar | 1 | 1 |
| *Asplenium scolopendrium* L. | Circumpolar | 1 | 1 |
| *Aconitum variegatum* L. *subsp. gracile* Gáyer | Central-European-Alpine | 1 | 1 |
| *Valeriana tripteris* L. | Central-European-Alpine | 1 | 1 |
| *Moehringia muscosa* L. | Central-European | 1 | 1 |
| *Tragopogon floccosus* Waldst. & Kit. | Pannonian | 1 | 1 |
| *Linum tenuifolium* L. | Pontic-Mediterranean | 1 | 1 |
| *Ajuga laxmannii* (Murray) Benth. | Pontic-Pannonian | 1 | 1 |
| *Coronilla emerus* L. | South-Mediterranean | 1 | 5 |
| *Lycopodium clavatum* L. | Cosmopolitan | 1 | 1 |
| *Androsace maxima* L. | Eurasian | 1 | 1 |
